# A cross-sectional study of current work ability after radical prostatectomy

**DOI:** 10.1186/s12894-020-0579-9

**Published:** 2020-01-31

**Authors:** Alv A. Dahl, Bjørn Brennhovd, Sophie D. Fosså, Karol Axcrona

**Affiliations:** 1 National Advisory Unit for Late Effects after Cancer Therapy, Oslo University Hospital, Radiumhospitalet, P. O. Box 4953, Nydalen, 0424 Oslo, Norway; 2 University of Oslo, Oslo, Norway; 3 Department of Urology, Oslo University Hospital, Radiumhospitalet, Oslo, Norway; 4 Department of Urology, Akershus University Hospital, Lørenskog, Norway

**Keywords:** Work ability, EPIC-26, Prostatic neoplasm, Robot-assisted laparoscopic prostatectomy, Depression

## Abstract

**Background:**

Work ability represents a person’s subjective assessment of current ability to work compared to his lifetime best. Since many men with prostate cancer are retired, work ability represents a more relevant work measure than employment status. The primary aim was to examine the prevalence of men who had high versus moderate/poor current work ability compared to their lifetime best work ability at a mean of 3.0 years after robot-assisted laparoscopic prostatectomy. The secondary aim was to study variables associated with moderate/poor work ability at survey.

**Methods:**

This is a questionnaire-based study of men who had robot-assisted laparoscopic prostatectomy at Oslo University Hospital, Radiumhospitalet between January 2005 and August 2010. Among them 777 responded (79%), 730 reported on current work ability, socio-demographic data, somatic and mental health, and typical adverse effects (the EPIC-26) after prostatectomy. High versus moderate/poor work ability was the primary outcome**.** Descriptive statistics and logistic regression analyses were applied.

**Results:**

The mean age of the sample at survey was 65.5 years (SD 5.9). At survey 42% of the sample reported moderate/poor current work ability and 58% reported high work ability. In multivariable analysis older age at survey, low basic education, comorbidity, poor self-rated health, presence of depression and low EPIC-26 hormonal domain score remained significantly associated with moderate/poor work ability.

**Conclusions:**

Current work ability is a useful measure for the working capacity particularly of retired men. Socio-demographic, cancer-related, health, psychological and typical adverse effect variables were significantly associated with moderate/poor current work ability after robot-assisted laparoscopic prostatectomy, and several health and psychological variables are amenable to identification and treatment by health care providers.

## Background

In Norway official retirement age is 67 years, but this age varies somewhat according to professions. The retirement age also varies in different countries, just as does the income granted after retirement. Many men are diagnosed and treated for prostate cancer (PCa) after retirement age. For them the official categories of employment such as paid work, unemployed, disability pension, or being on sick-leave, are not relevant as descriptors of their remaining working capacity. Many elderly PCa survivors use their considerable current work ability for charity, family, hobbies, or social organizations making valuable, often unpaid contributions to society. Major treatment modalities for localized PCa are radical prostatectomy, radiotherapy, and active surveillance. Treatment is chosen according to risk group, age, and general health as well as preferences of the patients. Major side effects after radical prostatectomy are urinary incontinence, erectile dysfunction, reduced feelings of masculinity and self-esteem, and partner conflicts often affecting the patients’ quality of life negatively [1]. At major urological centers radical prostatectomy is mainly performed as robot-assisted laparoscopic prostatectomy (RALP) as in this study.

A review of work-related self-report measures for cancer survivors, lists only one measure for ability to work: The Work Ability Index (WAI) [2]. The WAI was developed by a Finnish research group with a 24 items version [3–5]. Further research has demonstrated that the single item of current work ability score (WA) on a numeric scale from zero (‘Currently not able to do work’) to 10 (‘Work ability as previous life-time best’) from the WAI instrument showed strong correlation with the total score of the WAI short version and the psychometrics of rating WA in this way is well documented [6]. Therefore, we used a dichotomy of the current WA score as outcome variable of this study.

Studies of current WA among PCa survivors are few. Taskila et al. [7] reported that the mean current WA score of Finnish PCa survivors (*N* = 46) was 8.0, but they had no controls. A Nordic study reported a mean of 7.6 for current WA score in PCa survivors (*N* = 112) versus 8.3 among cancer-free controls (*p* < 0,01) [8]. Dahl et al. [9] studied the current WA of Norwegian PCa survivors (*N* = 563) who were *still active in the workforce* less than 3 years after radical prostatectomy (RP). They dichotomized the current WA scores into poor/moderate (0–7 points), good (8–9 points), and excellent (10 points), and found that 24% of the survivors belonged to the moderate/poor WA category. They found that men with post-RP radiotherapy or hormone treatment, urinary leakage, age above 65 years, or comorbidity significant more frequently belonged to the poor/moderate WA category compared to men of better WA categories [9].

These cross-sectional studies of current WA in men after RP are in need of expansion and replication since two of the studies have quite small samples. On this background, we analyzed data from a mailed questionnaire study of Norwegian men treated with RALP at our comprehensive cancer center. First, we identified the prevalence of PCa survivors with high versus moderate/poor current WA. Second, we examined variables significantly associated with moderate/poor WA. Given the available evidence, we did not expect an association among WA and PCa characteristics and treatment variables.

## Methods

### Sample and treatment

Between January 2005 and August 2010, 988 men underwent RALP as primary treatment for PCa at Oslo University Hospital, Radiumhospitalet. By March 2011 six men had died, and a questionnaire was mailed to the remaining 982 patients, and 777 responded (79% response rate). An attrition analysis between the respondents and the 205 non-respondents showed no significant differences on PCa-related and surgical variables except that the non-respondents were younger and had higher Clavien sum scores for operative complications [10]. Forty-seven responders were not included in our analysis due to incomplete data on current WA, leaving 730 men as our study sample.

Patients were operated with the same technique during all years (2005–2010), the Vattikutti technique, also described and published by our group [10, 11]. The principles of this technique are basically used in the majority of centers operating RALP. The follow-up of patients has been consistent and equal for all patients in the study period and thereafter.

### Measures

*Current WA* compared to the lifetime best WA was self-rated on a 10-point numerical rating as previously decribed [3–5]. We dichotomized the current WA scores into high WA (score 8–10) versus moderate/low WA (score 0–7).

#### Anxiety and depression

The Hospital Anxiety and Depression Scale (HADS) covers the last 7 days. Both the depression and the anxiety subscales have 7 items scored on a 4-point scale from 0 (‘not present’) to 3 (‘considerable’), with subscale sum scores ranging from 0 to 21. The cut-off scores for clinical anxiety and depression is a sum score ≥ 8 [11, 12]. Cronbach’s coefficient alpha was 0.85 for the anxiety and 0.79 for the depression subscale.

*Neuroticism* was self-rated on an abridged version of *The Eysenck Personality Inventory (EPQ)* for trait affects with six items each scored as present (1) or absent (0) [13]. The sum score ranged from 0 to 6, and was dichotomized into high (sum score 3–6) and low neuroticism (sum score 0–2) according to Grav et al. [14]. Cronbach’s alpha was 0.78.

#### Typical AEs

The EPIC-26 is a self-report instrument for rating of typical AEs of the *last 4 weeks* covering the urinary, bowel, sexual, and hormonal domains after PCa treatment. While the urinary and sexual domains cover both function and bother, the bowel and hormonal ones cover only bother. The scores are converted from 0 (worst) to 100 (best) and group means are calculated [15, 16].

*Among PCA-related variables* pre-treatment risk groups were defined according to D’Amico et al. [17]. Biochemical PSA relapse, post-RP radiotherapy, and hormone treatment after RALP were self-reported, and defined as *PCa treatment failure.*

#### Partnership status

Men were either married or cohabiting or were not living with a partner. *Non-employed status* concerned men who were without paid work or pensioned. *Low level of education* was defined as ≤12 school years completed versus high level (> 12 years). *Comorbidity* was based on self-report of stroke, diabetes, chronic obstructive lung diseases, liver disease, arthrosis, rheumatic diseases (all 1 point), and kidney disease (2 points) based on illness points according to Charlson et al. [18].

### Statistical analysis

Descriptive statistics were performed with chi-square tests for categorical variables and independent sample t-tests for continuous variables, but with Mann-Whitney U-tests in case of skewed distributions. One-sample t-tests were used to compare the current WA mean score of our sample with that of other published samples.

To find the *p*-value adjusted for age, we used multivariate logistic analyses for categorical data, and multilinear linear analyses for continuous data. These statistical procedures were performed for each of the age-relevant independent variables with Low/moderate WA versus High WA (reference) as dependent variable.

Univariate and multivariable logistic regression analyses with relevant independent variables and moderate/low WA as outcome variable and high WA as reference were performed. The strength of associations was described by odds ratios (ORs) with 95% confidence intervals (95%CI). The level of significance was set at *p* < 0.05, and all tests were two-sided. Data analyses were performed with IBM SPSS version 25.0 for PC (IBM, Armonk, NY).

## Results

### Characteristics of the total sample

The mean age of the sample at surgery was 62.5 years (SD 5.7). Of the total sample 23% belonged to the low risk, 41% to the intermediate, and 36% to the high risk group, and positive margins were observed in 28%. Only nerve-sparing was significantly less frequent in the moderate/poor compared to the high WA group (Table 1).

**Table 1:** Cancer-related data of the work ability groups at surgery

Variables	Moderate/poor work ability (*N* = 309)	High work ability (*N* = 421)	*p*-value	Total sample (*N* = 730)
*Age at surgery, mean (SD)*	63.5 (5.6)	61.8 (5.7)	**< 0.001**	62.5 (5.7)
*Clinical stage N (%)*			0.74	
T1 – T2a	198 (64)	281 (67)		479 (66)
T2b	40 (13)	49 (11)		89 (12)
T3	71 (23)	91 (22)		162 (22)
*Gleason scores, N (%)*			0.31	
6	112 (36)	175 (42)		287 (39)
7	152 (49)	185 (44)		337 (46)
8–10	45 (15)	61 (14)		106 (15)
PSA. mean (SD)	13.5 (15.5)	12.3 (17.5)	0.32	12.8 (16.7)
*D’Amico risk categories, N (%)*			0.16	
Low	61 (20)	103 (24)		164 (23)
Intermediate	125 (40)	176 (42)		301 (41)
High	123 (40)	142 (34)		265 (36)
*Positive surgical margins, N (%)*	95 (31)	1|12 (27)	0.22	207 (28)
*Nerve sparing, N (%)*			**< 0.001**	
Bilateral	61 (20)	126 (30)		187 (26)
Unilateral	133 (43)	195 (46)		328 (45)
None	115 (37)	100 (24)		216 (29)
*Patological stage, N (%)*			0.24	
pT2a – pT2c	145 (47)	216 (51)		361 (49)
pT3a – pT3b	164 (53)	205 (49)		369 (51)

The mean age of the sample at survey was 65.5 years (SD 5.9). At survey 42.3% (95%CI 38.7–45.9%) of the sample reported moderate/poor current WA and 57.7% (95%CI 54.1–61.3%) reported high WA.

Of the sample 21% had experienced treatment failure at the survey which took place at a mean of 3.0 years (SD 1.4) after surgery (Table 2).

**Table 2:** Demographic, and health findings of the work ability groups at survey

Variables	Moderate/poor work ability (*N* = 309)	High work ability (*N* = 421)	*p*-value	Total Sample (*N* = 730)
Age at survey, mean (SD)	66.4 (5.8)	64.8 (5.8)	**< 0.001**	65.5 (5.9)
Time RALP-survey (years), mean (SD)	3.0 (1.4)	3.0 (1.5)	0.73	3.0 (1.4)
Treatment failure, N (%)	74 (24)	80 (19)	0.11	154 (21)
*Basic education, N (%)*			**< 0.001**	
> 12 years	158 (51)	289 (69)		447 (61)
≤ 12 years	151 (49)	132 (31)		283 (39)
Paired relationship, N (%)	276 (89)	392 (93)	0.04*	668 (92)
*Employment status, N (%)*			< 0.001*	
Working full-time	53 (17)	228 (55)		281 (38)
Working part-time	25 (8)	35 (8)		60 (8)
Retired	149 (49)	142 (34)		291 (40)
Sick-leave/rehabilitation	18 (6)	5 (1)		23 (3)
Disability pension	48 (15)	2 (0)		50 (8)
Unspecified	16 (5)	9 (2)		25 (3)
*Work ability, mean (SD)*	5.4 (1.8)	8.8 (0.8)	< 0.001*	7.4 (2.1)
*Comorbidity index, N (%)*			< 0.001*	
0 point	191 (62)	345 (82)		536 (73)
1 point	85 (28)	60 (14)		145 (20)
≥ 2 points	33 (10)	16 (4)		49 (7)
Poor self-rated health, N (%)	70 (23)	14 (3)	< 0.001*	84 (12)
High neuroticism, N (%)#	113 (37)	51 (12)	< 0.001*	164 (23)
Clinical anxiety, N (%)#	43 (14)	18 (4)	< 0.001*	61 (8)
Clinical depression, N (%)#	42 (14)	8 (2)	< 0.001*	50 (7)

*Adjusted for age at survey #High neuroticism = EPQ Neuroticism score ≥ 3; Clinical anxiety = HADS Anxiety sum ≥8; Clinical depression = HADS Depression sum ≥8,

### Comparisons of the moderate/poor and the high WA groups at survey

At survey the moderate/poor WA group had a significantly lower proportion in paired relationships, a lower proportion was still working full-time, and a higher proportion was retired compared to the high WA group. The poor/moderate WA group also had higher rates of comorbidity, poor self-rated health, high neuroticism, and men with anxiety or depression (Table 2).

The moderate/poor WA group had poorer mean function and bother score on all EPIC-26 issues compared to the high WA group (Table 3).

**Table 3:** EPIC-26 mean and standard deviation (SD) adverse effect findings of the work ability groups at survey

Variables	Moderate/poor work ability (*N* = 309)	High work ability (*N* = 421)	*p*-value	Total sample (*n* = 730)
*Urinary domain, mean (SD)*	70.3 (24.0)	80.1 (21.1)	< 0.001*	76.1 (22.9)
Incontinence subscale	65.2 (28.4)	74.5 (26.7)	< 0.001*	70.5 (27.8)
Irritation/obstruction subscale	78.0 (26.8)	86.1 (21.9)	< 0.001*	82.7 (24.4)
Overall urinary problem	68.4 (29.8)	79.8 (27.3)	< 0.001*	76.6 (22.0)
*Bowel domain, mean (SD)*	86.1 (20.5)	93.0 (15.0)	< 0.001*	90.1 (17.8)
Overall bowel problem	87.9 (22.1)	94.2. (15.2)	< 0.001*	89.2 (20.5)
*Sexual domain, mean (SD)*	26.6 (25.8)	35.5 (28.3)	0.001*#	30.8 (27.7)
Erection ability	17.6 (26.7)	25.0 (29.8)	0.011*#	21.8 (28.8)
Difficulty with orgasm	32.2 (32.3)	42.5 (33.0)	0.002*#	38.1 (33.1)
Firmness of erections	34.4 (39.4)	45.9 (40.8)	0.004*#	41.0 (40.6)
Reliability of erections	25.6 (37.9)	33.7 (40.4)	0.06*#	30.3 (39.6)
Sexual function	18.9 (26.1)	27.4 (29.6)	0.001*#	23.8 (28.5)
Overall sexual problem	30.7 (31.4)	38.4 (33.2)	0.002*#	35.2 (32.1)
*Hormonal domain; mean (SD)*	74.2 (24.7)	90.7 (16.6)	< 0.001*	83.8 (21.9)
Hot flashes	89.8 (23.4)	95.9 (15.1)	< 0.001*	93.6 (19.0)
Breast problems	93.8 (18.4)	98.2 (9.3)	< 0.001*	96.4 (13.9)
Depression	68.0 (31.7)	88.4 (22.1)	< 0.001*	81.1 (27.6)
Lack of energy	59.1 (34.0)	86.4 (22.6)	< 0.001*#	76.3 (30.3)
Weight change	79.9 (28.9)	92.8 (18.4)	< 0.001*	88.8 (23.4)

*Adjusted for age at survey; # Mann-Whitney U-test

### Univariate and multivariable analyses

The univariate analyses (not adjusted for age at survey like in Table 3) with moderate/poor WA as dependent variables mostly confirmed the significant between-group differences reported in Tables 3 and 4. Older age at survey, low basic education, comorbidity, poor self-rated health, high neuroticism, presence of depression and low EPIC-26 hormonal domain score remained significantly associated moderate/poor WA in the multivariable analysis (Table 4).

**Table 4:** Univariate and multivariable logistic regression analyses of independent variables in relation to moderate/poor work ability (high as reference) as dependent variable

Variables	Univariate analyses			Multivariable analysis		
	OR	95%CI	*p*- value	OR	95%CI	*p*- value
*Nerve-sparing*			**< 0.001**			0.06
Bilateral (reference)	1.00	–	–	1.00	–	–
Unilateral	2.38	1.58–3.27	**< 0.001**	1.68	1.02–2.76	**0.04**
None	1.41	0.97–2.05	0.08	1.09	0.69–1.72	0.71
Age at survey	1.06	1.03–1.08	**< 0.001**	1.08	1.04–1.12	**< 0.001**
Low basic education	2.09	1.55–2.84	**< 0.001**	1.84	1.29–2.64	**0.001**
Non-paired relationship	1.62	0.96–2.02	0.07	–	–	–
*Comorbidity*			**< 0.001**			**< 0.001**
0 point (reference)	1.00	–	–	1.00	–	**–**
1 point	2.56	1.76–3.72	**< 0.001**	2.39	1.54–3.62	**< 0.001**
≥ 2 points	3.73	2.00–6.94	**< 0.001**	3.05	1.46–6.35	**0.003**
Poor self-rated health	8.47	4.67–15.37	**< 0.001**	5.66	2.76–11.60	**< 0.001**
High neuroticism^a^	4.18	2.88–6.07	**< 0.001**	1.70	1.01–2.85	**0.045**
Clinical anxiety^a^	3.62	2.04–6.71	**< 0.001**	1.62	0.73–3.60	0.23
Clinical depression^a^	8.12	3.75–17.57	**< 0.001**	4.37	1.60–11.97	**0.004**
Urinary domain	0.98	0.97–0.99	**< 0.001**	0.99	0.98–1.00	0.09
Bowel domain	0.98	0.97–0.99	**< 0.001**	1.00	0.99–1.01	0.96
Sexual domain	0.99	0.98–0.99	**< 0.001**	1.00	1.00–1.01	0.51
Hormonal domain	0.96	0.95–0.97	**< 0.001**	0.98	0.97–0.99	**< 0.001**

^a^High neuroticism = EPQ Neuroticism score ≥ 3; Clinical anxiety = HADS Anxiety sum ≥8; Clinical depression = HADS Depression sum ≥8

## Discussion

### Summary of main findings

In our sample of Norwegian men treated with RALP at a mean of 3 years earlier and with mean age of 65.5 years, 42% reported moderate/poor current WA and 58% reported high WA compared to their lifetime best. Our hypothesis was supported since only nerve-sparing among the PCa-related variables was associated with moderate/poor WA, while several demographic, health, psychological, and AEs variables showed such associations in bivariate analyses. Such variables also remained significant in the multivariable analyses.

### The usefulness of the WA measure

Current WA compared to the lifetime best is a subjective concept rated by the patients, and thereby could be considered as a patient-reported outcome measure (PROM). WA is often divided into three components: physical, mental, and social, and in modern work life there has been a change of focus from the physical to social aspect [19]. We could speculate on when in men’s life is their WA at “the lifetime best”? Physically the answer is young adult life, but with increased emphasis on the mental and social aspects “lifetime best” could occur in midlife due to training and experience. Our main point here is that the comparison between current WA and “lifetime best” do not necessarily imply a long-time span.

From Table 2 we see that 38% of our sample still are working full-time, while 40% are retired, so employment status does do not correlate highly with current WA in our sample. In contrast, current WA in retired men is used for many purposes both private, in the family, and in society. Rather than ignoring the WA of retired men, the WA measure shows the considerable work resources in this elderly group of men treated with RALP. We found that the full-time workers have a much higher level of current WA than those who are in part-time employment or retired. Such a finding is not unexpected, but it does raise the question of whether the PCa cancer survivors’ current work ability is enhanced by those continuing full-time work or whether their ability to engage in full-time work is influenced by the current WA. The general attitude of the Norwegian government is that persons who have reasonable WA should stay in their jobs as long as possible., and the GPs following these men should consider tehri optimal current WA.

Psychometric objections have been made for measuring current WA with only one item, but high correlation with the full WA score based on 24 items has been demonstrated [6]. A major objection to the current WA concept is the lack of population-based reference data both continuous and categorical. The validity of the findings if the Finnish Health 2000 Survey are doubtful in relation to recent studies from other countries [19].

### Comparisons with previous WA findings in men with PCa

In our sample of PCa survivors, the current WA mean score was 7.4 (SD 2.1) (Table 2). A Finnish sample with 46 survivors had a mean of 8.0, and a Nordic study with 112 survivors reported a mean of 7.6 for current WA. These mean scores were significantly higher than the mean current WA score of our study (one sample t-test *p* < 0.01).

The Dahl et al. study [8] included only men who were still active in the workforce after RP, so their high mean current WA score of 8.6 (SD 0.5) should be interpreted according to this premise. Being still in the workforce is also the explanation that their sample had 76% of men with high current WA in contrast to 58% in our sample including all sorts of work statuses (*p* < 0.001).

### New findings

Not surprisingly, both the Finnish [7] and the Norwegian [9] studies found that both older age and comorbidity was significantly associated with reduced current WA score. In addition, the Finnish study found low education, and the Norwegian post-operative urinary leakage also were significantly associated with reduced current WA. Our study supported these findings, but in addition, we found that all domains of typical AEs after RALP were significantly associated with moderate/poor WA in bivariate analyses. Interestingly, of the four EPIC-26 domains scores (urinary, bowel, sexual, and hormonal) entered into the multivariable analysis, only the hormonal domain score remained significantly associated with moderate/poor WA. This could be associated with hormone treatment due to treatment failure, but also be due to depression in general since lack of energy is a central symptom of the depressive syndrome.

### Clinical implications

We found that moderate/poor current WA was associated with several variables that should be amenable to diagnosis and treatment by health care providers, eventually improving current WA. Such variables were comorbidity, anxiety, and depression.

### Strengths and limitations

Strengths of our study are the considerable sample size of men treated with RALP for PCa; an attrition analysis supporting the external validity of our findings; and use of established instruments with good psychometric properties. One limitation of the study is the cross-sectional design, and that we thereby lack pre-treatment data of current WA. Prospective studies of current WA in men treated for PCa are needed in order to understand its influence on later WA. Reference data on current WA in older men is also needed. Another limitation was that the patients provided self-report data of the comorbidities rather than collecting such data from their medical records and their general practitioners.

## Conclusions

Current WA is a useful measure for the working capacity of retired men. In our sample, 42% of the PCa survivors reported moderate/poor current WA and 58% reported high WA, while 38% were in full-time and 8% in part-time work. In multivariable analysis older age, low basic education, comorbidity, poor self-rated health, high neuroticism, increased level of depression, and lower EPIC-26 domain score. Several of these variables are amenable to identification and treatment by health care providers.

## Data Availability

According to Norwegian data legislation, the data of this study cannot be made generally available. Requests may be made to the first author.

## Abbreviations

CI: Confidence Interval
EPIC-26: The Expanded Prostate Cancer Composite-26
HADS: The Hospital Anxiety and Depression Scale
PCa: Prostate Cancer
PSA: Prostate Specific Antigen
RALP: Robot-Assisted Laparoscopic Prostatectomy
RP: Radical Prostatectomy
SD: Standard Deviation
WA: Work Ability
WAI: The Work Ability Index
